# A Novel Fluorescent Aptamer Sensor with DNAzyme Signal Amplification for the Detection of CEA in Blood

**DOI:** 10.3390/s23031317

**Published:** 2023-01-24

**Authors:** Qingmin Wei, Huakui Huang, Shulong Wang, Fa Liu, Jiayao Xu, Zhihui Luo

**Affiliations:** 1 Guangxi Key Lab of Agricultural Resources Chemistry and Biotechnology, College of Chemistry and Food Science, Yulin Normal University, 1303 Jiaoyudong Road, Yulin 537000, China; 2 Yulin Campus, Guangxi Medical University, 22 Shuangyong Road, Nanning 530021, China

**Keywords:** fluorescent aptamer sensor, carcinoembryonic antigen, signal amplification, DNAzyme

## Abstract

Carcinoembryonic antigen (CEA) is a tumor-specific biomarker; however, its low levels in the early stages of cancer make it difficult to detect. To address the need for analysis of ultra-low-level substances, we designed and synthesized a fluorescent aptamer sensor with DNAzyme signal amplification and used it for the detection of CEA in blood. In the presence of the target protein, the aptamer sequence in the recognition probe binds to the target protein and opens the hairpin structure, hybridizes with the primer and triggers a polymerization reaction in the presence of polymerase to generate double-stranded DNA with two restriction endonuclease Nb.BbvCl cleavage sites. At the same time, the target protein is displaced and continues to bind to another recognition probe, triggering a new round of polymerization reaction, forming a cyclic signal amplification triggered by the target. The experimental results show that the blood detection with CEA has a high sensitivity and a wide detection range. The detection range: 10 fg/mL~10 ng/mL, with a detection limit of 5.2 fg/mL. In addition, the sensor can be used for the analysis of complex biological samples such as blood.

## 1. Introduction

Nucleic acid aptamers have advantages such as high specificity, easy synthesis and modification, low cost of synthesis, conformational tautological properties of molecular recognition and operational characteristics of nucleic acid tool enzymes [1,2], and have been widely used in the construction of various biosensors, such as fluorescence aptamer sensors, electrochemical aptamer sensors and colorimetric aptamer sensors. Among them, fluorescent aptamer sensors have become the most studied class of aptamer sensors due to their simplicity, rapidity and homogeneous analysis [3]. However, the sensitivity of conventional fluorescent aptamer sensors is generally low because the nucleic acid aptamer-to-target molecule binding ratio is generally 1:1, and each molecule of target can only trigger a change in the fluorescence signal of one molecule of probe. In order to improve the detection sensitivity of fluorescent aptamer sensors, a series of nucleic acid signal amplification strategies have been proposed by national and international researchers in recent years, such as enzyme cycle cutting reactions [4,5], cyclic chain substitution reaction [6], rolling loop amplification reaction, hybrid chain reaction [7] and DNA cyclic self-assembly reactions [8], etc. With the introduction of these nucleic acid signal amplification strategies, the detection sensitivity of fluorescent aptamer sensor has been greatly improved, but it still can’t meet the needs of the detection of some ultra-low content substances, especially disease markers. Therefore, it is still necessary to construct simple, rapid and more sensitive fluorescent aptamer sensors.

The exponential amplification reaction is an emerging isothermal cyclic amplification technique for nucleic acids that combines DNA polymerase-catalyzed reactions and restriction cleavage endonuclease-based cleavage reactions to enable exponential amplification of specific nucleic acid sequences under constant temperature conditions [9,10,11]. The isothermal exponential amplification reaction has the advantages of fast reaction rate and high amplification efficiency, and has been widely used for the detection of nucleic acids, organic small molecules, proteins, cells and other targets. For example, Zhang et al. constructed a sensing platform of single microbeads based on isothermal exponential amplification reaction, which can achieve ultra-sensitive detection of micro RNAs at the single molecule level [12]. For example, isothermal exponential amplification reaction can also be used for fluorescence [13], electrochemical analysis and chemiluminescence analysis of microRNAs [14]. In addition, using isothermal exponential amplification reaction amplification techniques, combined the advantages of nucleic acid aptamers with fluorescence [15,16], colorimetric [17] and surface-enhanced Raman detection techniques [18], aptamer sensors can be developed for protein, organic small molecule and cellular detection. Although isothermal exponential amplification reaction amplification technique has made a series of advances in biosensing research, its detection sensitivity is still unsatisfactory when used to construct aptamer sensors, especially fluorescent aptamer sensors, and needs to be further improved.

Deoxyribonuclease (DNAzyme) is a catalytically active single-stranded DNA obtained by in vitro screening [19]. Compared with traditional proteases, DNAzyme has the advantages of good thermal stability, high specificity, easy synthesis and modification, low cost and high catalytic efficiency. All these advantages of DNAzyme makes it promising for applications in biosensing research. In particular, metal ion-dependent DNAzyme has been widely used for sensitive analysis of targets such as metal ions [20], small molecules [21], proteins [22,23], nucleic acids [24] and bacteria [25] due to its ability to catalyze cyclic cleavage of its substrates with signal amplification. For example, Hu et al. proposed a DNAzyme-based enzyme-linked immunosorbent assay for multiplex immunoassay of proteins. This immunoassay has the advantages of high throughput, simplicity and rapidity, and was successfully applied to the simultaneous detection of three different sources of immunoglobulin G [26]. Cheng et al. developed an electrochemical luminescence strategy for the detection of Mg^2+^ using the principle of DNAzyme regulating electrochemical luminescence signal conversion [27]. Furthermore, Wei et al. constructed a colorimetric sensing method for the highly sensitive detection of proteins using the principle of target-mediated proximity hybridization to activate DNAzyme [28]. Although DNAzyme has made rapid developments in improving the performance of biosensors, it is still not suitable for the analysis of ultra-low-level substances.

CEA is a tumor-specific biomarker that is commonly found in plasma, pleural fluid and ascites, and is currently detected clinically mainly by blood, which is relatively low; therefore, it is difficult to detect it precisely. In this paper, to address the needs for the analysis of ultra-low content substances of CEA, a novel ultra-sensitive fluorescent biosensor was constructed for CEA protein analysis by combining the advantages of isothermal exponential amplification reaction, DNAzyme signals amplification technique and nucleic acid aptamer. This method uses the combination of target and nucleic acid aptamer to trigger an isothermal exponential amplification reaction to synthesize a large number of DNAzyme sequences, and then the DNAzyme sequences catalyze the cyclic cleavage of their molecular beacon substrates in the presence of Mg^2+^ to amplify the fluorescence signal, thus realizing target detection. The detection sensitivity of the method can be significantly improved due to the introduction of isothermal exponential amplification reaction and the dual signal amplification technology of DNAzyme. The method was validated using CEA as the target analyte, and the results show that the sensor has an ultra-high sensitivity and a wide linear range, with a detection range of 10 fg/mL~10 ng/mL and the limit of detection (LOD) reached 5.2 fg/mL for CEA. The results show that the sensor has high specificity and can be used to detect the specificity of complex biological samples.

## 2. Materials and Methods

### 2.1. Materials

Thrombin (Tb), human serum albumin (HSA), bovine serum albumin (BSA) and ethidium bromide (EB) were bought from Sigma-Aldrich Company (St. Louis, MO, USA). Recombinant human vascular endothelial growth factor-165 (VEGF165) was bought from the Invitrogen Company (Waltham, MA, USA). Nb.BbvCI, Polymerase Klenow fragment exo- (KF), KF buffer solution and TBE buffer solution were bought from the New England Biolabs Company (Ipswich MA, USA). DNA marker, deoxynucleotide (dNTPs), tromethamine (Tris) and CEA were purchased from Sangon Bioengineering Co., Ltd. (Shanghai, China). Other chemical reagents are analytically pure. The detection buffer solution (pH 8.0) was composed of 20 mM HEPES, 100 mM NaCl, 20 mM MgCl_2_ and 20 mM KCl. Water used in the experiment was 18.2 MΩ•cm ultrapure water.

The synthesized nucleotide chains used in this experiment were synthesized by Invitrogen Company (USA) and purified by high performance liquid chromatography, and their sequences are listed in Table 1. The italic bold parts are the aptamer sequences of CEA.

### 2.2. Apparatus

Experimental apparatus includes LS-55 fluorospectro photometer (PerkinElmer Company, Waltham, MA, USA), DYY-8C electrophoresis instrument power supply, Omega 16ic gel imaging analysis system (ULTRA-LUM, USA), DYCZ-24DN double vertical electrophoresis tank, FA604 electronic balance, PHSJ-4A precision pH meter, DF-101S constant temperature heating magnetic agitator, KQ5200B ultrasonic cleaner, H1650-W high speed microcentrifuge.

### 2.3. Serum Sample Pretreatment

Three serum samples of normal subjects and three serum samples of colon cancer patients were collected from the Fifth People’s Hospital of Guilin. All serum samples were diluted 100 times with pH 8.0, 20 mM HEPES, 100 mM NaCl, 20 mM MgCl_2_ and 20 mM KCl buffer solution, and then analyzed and detected.

### 2.4. Fluorescence Measurements

All fluorescence detection experiments were carried out on LS-55 fluorescence spectrophotometer. The sample solution was excited at 485 nm and detected at 520 nm, and the excitation and emission slit widths were set to 10 nm. All experiments were repeated three times, and each sample solution was detected five times in parallel.

### 2.5. Feasibility Study

The six samples used to verify the feasibility of the experimental principle were prepared as follows: (1) Preparation of sample a: 10 μL of CEA (20 pg/mL) solution was added to a mixture (40 μL) containing 100 nM of H-probe, 100 nM of primer-3, and incubated at 37 °C for 60 min, followed by the addition of substrate MB containing 300 nM and 20 mM Mg^2+^; (2) Preparation of sample b: the same procedure as sample, except 10 μL of detection buffer solution was used instead of CEA solution; (3) Preparation of sample c: 10 μL of CEA (20 pg/mL) solution was added to the mixture containing 100 nM H-probe, 100 nM primer-3, 5U KF, 25 μM dNTPs (40 μL) and incubated at 37 °C for 60 min, then 150 μL HEPES detection buffer solution containing 300 nM substrate MB and 20 mM Mg^2+^ was added for another 60 min to obtain sample c; (4) Preparation of sample d: except 10 μL of detection buffer solution was used instead of CEA solution; (5) Add 10 μL of CEA (20 pg/mL) solution to a mixture of 100 nM H probe, 100 nM primer-3, 5U KF, 5U Nb.BbvCI, 25 μM dNTPs (40 μL), and incubate at 37 °C for 60 min, then add 300 nM of substrate MB and 20 mM Mg^2+^. The sample e was obtained by adding 150 μL of HEPES detection buffer solution containing 300 nM MB and 20 mM Mg^2+^ for another 60 min; (6) Preparation of sample f: the procedure was the same as that for sample e except 10 μL of detection buffer solution was used instead of CEA solution. The obtained sample solution was used for fluorescence detection, and the change of the fluorescence value of the solution was monitored.

### 2.6. Fluorometric Detection of CEA

The sample solution was incubated for 60 min at 37 °C with 10 μL of CEA solution at different concentrations in a mixture of 100 nM H probe, 100 nM primer-3, 5U KF, 5U Nb.BbvCI, 25 μM dNTPs (40 μL), and 150 μL HEPES buffer solution containing 300 nM substrate MB and 20 mM Mg^2+^ for another 60 min. The final sample solution was added to a quartz cuvette for fluorometric detection.

### 2.7. Gel Electrophoresis Measurement

The sample preparation process for gel electrophoresis analysis was as follows: (1) Sample 1 preparation: 2.0 μM nucleic acid-based hairpin probe H solution for sample 1; (2) Sample 2 preparation: 2.0 μM auxiliary primer-3 probe solution for sample 2; (3) Sample 3 preparation: H probe (2.0 μM) and auxiliary primer-3 (2.0 μM) probe were mixed, and the resulting BbvCI (2U) was reacted at 37 °C for 60 min, and the resulting solution was sample 4; (3) Preparation of sample 3: the H probe (2.0 μM) and the auxiliary primer-3 (2.0 μM) probe were mixed, and the resulting solution was sample 3; (4) Preparation of sample 4: the mixture of H probe (2.0 μM), primer-3 (2.0 μM), dNTPs (400 μM) and KF (2U), Nb.BbvCI (2U) was added at BbvCI (2U) at 37 °C for 60 min, and the resulting solution was sample 4; (5) sample 5 was prepared by adding CEA solution (20 ng/mL) containing H probe (2.0 μM), pimer-3 (2.0 μM), dNTPs (400 μM) and KF (2U), Nb. was added to a mixture of H probe (2.0 μM), auxiliary primer-3 (2.0 μM), dNTPs (400 μM) and KF (2U), and reacted at 37 °C for 60 min, and the resulting solution was sample 5; (6) preparation of sample 6: CEA solution (20 ng/mL) was added to a mixture of H probe (2.0 μM), auxiliary primer-3 (2.0 μM), dNTPs (400 μM), and KF (2U), and the resulting solution was sample 5; (6) preparation of sample 6: CEA solution (20 ng/mL) was added to a mixture of H probe (2.0 μM), auxiliary BbvCI (2U) at 37 °C for 60 min, and the resulting solution was sample 6; (7) preparation of sample 7: 2.0 μM DNA-1 solution was sample 7. Gel electrophoresis tests were performed in a DYCZ-24DN electrophoresis tank and DYY-8C electrophoresis instrument power supply, and 6 μL was fed on a 15% non-denaturing PAGE gel, and the gel was run in 1×TBE buffer and 80 V for 2 h. After staining with EB, the gel was photographed on a gel imaging system.

## 3. Results and Discussion

### 3.1. Analysis of CEA Detection Principle

**Figure 1:**
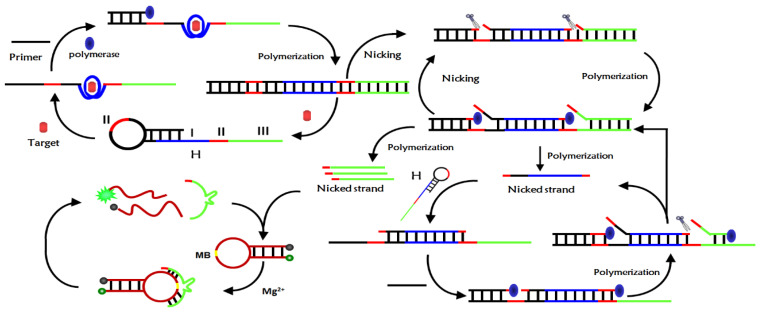
Detection principle of fluorescent aptamer sensor based on isothermal exponential amplification reaction and deoxyribonuclease signal amplification.

The detection mechanism of the novel fluorescent aptamer sensor based on isothermal exponential amplification reaction and DNAzyme was depicted in Figure 1. The sensor mainly consists of a hairpin nucleic acid probe H, primers, DNAzyme substrate MB, polymerase and restriction nucleic acid endonuclease Nb.BbvCI. The nucleic acid hairpin probe H consists of three main parts: region I was the aptamer sequence of the target, regions II was the Nb.BbvCI recognition sequence, region III was the Mg^2+^-dependent DNAzyme complementary sequence. The 3′ end of the H probe sequence contains a mismatch base to prevent polymerization when there was no target. When there was no target, H-probe and primer can exist stably and can’t initiate isothermal exponential amplification reaction to produce deoxyribozyme with catalytic activity. When the target was introduced into the sensor probe, the aptamer sequence of the H-probe specifically binds to the target and opens the hairpin structure of the H-probe. The primers hybridized with the open H-probe to initiate polymerization in the presence of polymerase and monomer to form long double-stranded DNA and replace the target substance at the same time.

The released target can combine with another H-probe to initiate a new round of polymerization, form a cyclic amplification of the target, and produce many double-stranded DNA. The resulting double-stranded DNA contains two cleavage sites of Nb.BbvCI, which are selectively cleaved by Nb.BbvCI to form two new polymerization sites. After polymerization, new double-stranded DNA was formed and DNA initiation chain and DNAzyme sequence were replaced. However, the resulting double-stranded DNA can be cleaved by Nb.BbvCI to form a cleavage-polymerization-replacement cycle, resulting in a large number of DNA initiation chains and DNAzyme sequences. The DNA initiation chain hybridizes with the H-probe and opens the hairpin structure of the H-probe, which reinitiates the polymerization to form double-stranded DNA and displace the DNA initiation strand. The released DNA initiation chain can hybridize with another H-probe, trigger a new round of polymerization, form DNA initiation chain cycle amplification and produce a large number of DNA double strands. These DNA double strands can also initiate the above cleavage-polymerization-replacement cycle reaction, resulting in exponential amplification. After exponential amplification, a large number of DNAzyme sequences were produced, which catalyze the cyclic cleavage of MB substrate probes in the presence of Mg^2+^, resulting in the separation of fluorescence groups and quenching groups, so the fluorescence signal was enhanced. The ultra-sensitive detection of CEA can be achieved by monitoring the change in fluorescence intensity of the system before and after the addition of the target CEA.

### 3.2. Feasibility Verification of CEA Detection

**Figure 2:**
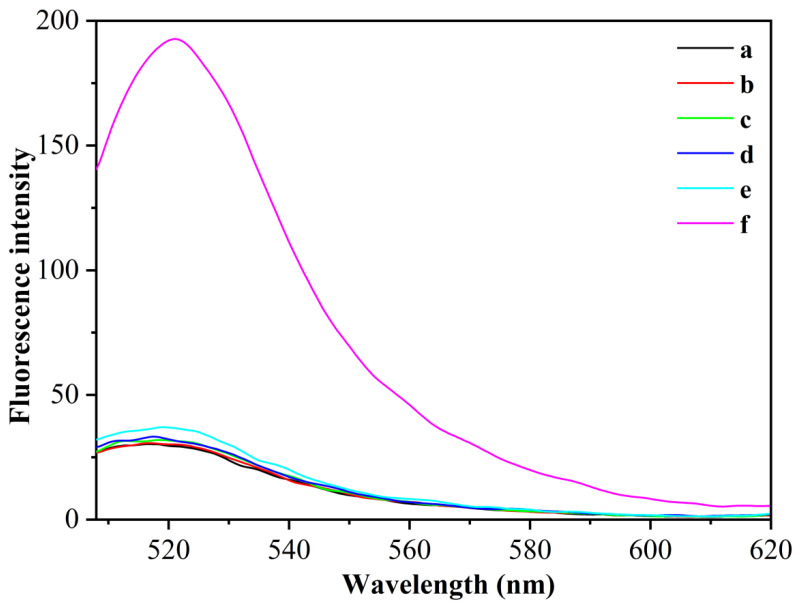
The fluorescence spectra with different sensing systems for the detection of CEA (1 pg/mL). (a) H/primer-3/MB; (b) H/primer-3/MB/CEA; (c) H/primer-3/MB/KF; (d) H/primer-3/KF/MB/CEA; (e) H/primer-3/KF/Nb.BbvCI; (f) H/primer-3/KF/Nb.BbvCI/CEA.

To verify the feasibility of the above fluorescence aptamer detection principle, we used different sensing systems for fluorescence detection of the same concentration of CEA (1 pg/mL) using CEA as the target analyte (Figure 2). When the H/primer-3/MB system was used, the fluorescence of the system hardly changed after the addition of the target CEA (curves a and b), which was due to the fact that in the absence of polymerase KF and the presence of Nb.BbvCI, CEA, after binding to the aptamer chain to open the hairpin nucleic acid probe, could not trigger the exponential amplification reaction to synthesize catalytically active DNAzyme sequences and could not cleave the MB substrate. When the H/primer-3/KF/MB system was used, the fluorescence of the system also did not change significantly after the addition of the target CEA (curves c and d), which was due to the fact that the target CEA could only undergo a cyclic polymerization reaction in this case, and could not generate DNAzyme sequences to cleave MB substrates. In contrast, when the H/primer-3/KF/Nb.BbvCI/MB system was used, the fluorescence of the system is significantly enhanced by the addition of the target CEA (curve f), which was due to the specific binding of the target CEA to the aptamer sequence in the H-probe, which can trigger the polymerase KF and Nt.BbvCI index-based amplification reaction and generate a large number of DNAzyme sequences; these DNAzyme sequences can hybridize with MB substrates and circularly cleave MB substrates, thus enhancing the fluorescence signal. The experimental results show that the simultaneous introduction of polymerase KF and Nt.BbvCI could significantly enhance the fluorescence detection signal of CEA, and this sensing probe could be used for the highly sensitive detection of CEA.

### 3.3. Gel Electrophoresis Characterization

**Figure 3:**
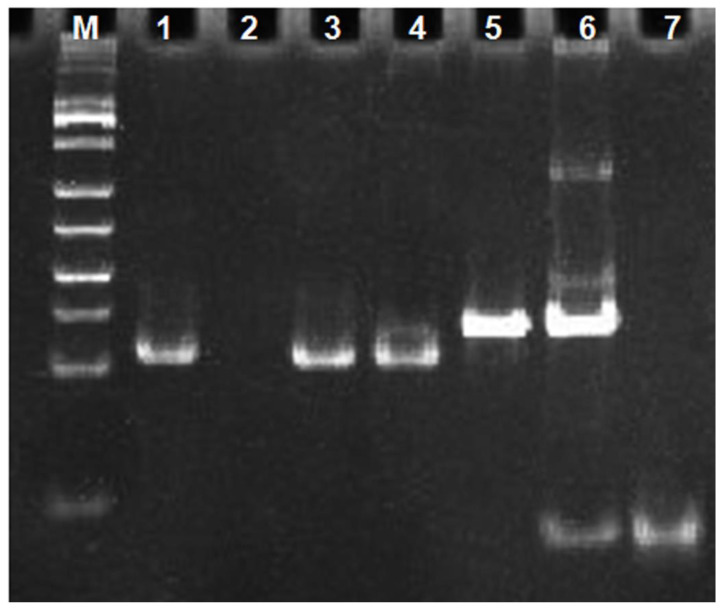
Gel electrophoresis analysis diagram. (M) DNA marker; (1) H; (2) primer-3; (3) primer-3/H; (4) H/primer-3/KF/Nb.BbvCI; (5) H/primer-3/KF/CEA (20 ng/mL); (6) H/primer-3/KF/Nb.BbvCI/CEA (20 ng/mL); (7) DNA-1.

To verify that target binding to nucleic acid aptamer sequences indeed triggers an exponential amplification reaction, we characterized the different systems by non-denaturing polypropylene amide gel electrophoresis experiments. The results are shown in Figure 3. The front 4 channels were DNA maker, H-probe, primer-3, and primer-3 with H-probe mixture, respectively. A clear band appears in channel 2, which was due to the presence of a double-stranded structure in the stem region of the H-probe. No obvious bands appear in channel 3 due to the short single-stranded DNA of eight bases for primer-3. No obvious bands appeared in channel 3, due to primer-3′s short, single-stranded DNA of eight bases. In channel 4, the same band as that in channel 2 appears, which is the band of H-probe. Compared to channel 4, channel 5 still shows only one band corresponding to the position in channel 4, indicating that the exponential amplification reaction was not triggered in the absence of the target CEA and could not generate the DNAzyme sequence. Channel 6 was a mixture of CEA, primer-3, H-probe and polymerase KF. Compared with channels 4 and 5, a new band with slower migration appears in channel 6 and the band of the H-probe became blurry, indicating that the polymerization reaction occurred and a relatively large molecular weight of double-stranded DNA had generated. Channel 7 was a mixture of CEA, primer-3, H-probe, polymerase KF and Nb.BbvCI. Compared with channel 6, the band of H-probe disappears in channel 7 and a band with a faster migration rate appears, which indicated that the exponential amplification reaction occurred and a large amount of single-stranded DNA had been generated. Channel 8 showed the synthetic DNA-1 sequence, which was the same as the single-stranded DNA sequence generated by the exponential amplification reaction. As can be seen in channel 8, the DNA-1 band appears in the same position as the band in channel 7 that produces a faster migration rate. This indicates that the DNAzyme sequence was generated in channel 7. The result of the gel electrophoresis experiments indicated that the target CEA can indeed trigger an exponential amplification reaction to generate DNAzyme sequences.

### 3.4. The Effect of Primer Chain Length on CEA Detection

**Figure 4:**
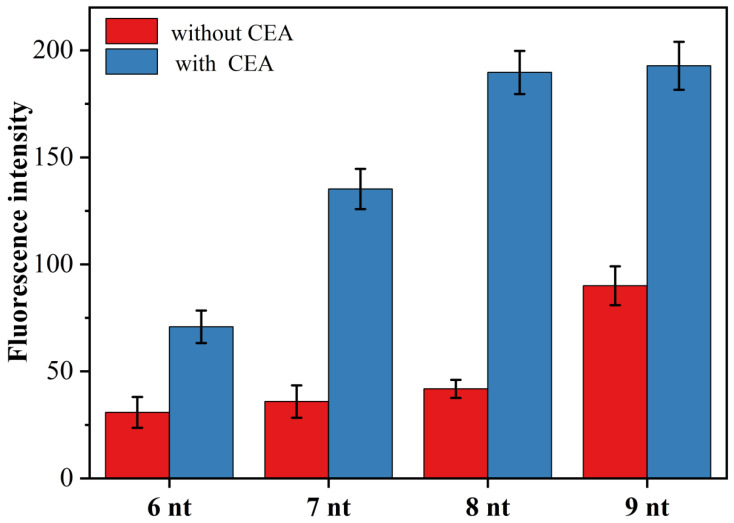
Effect of primer chain length on the fluorescence intensity of CEA (1.0 pg/mL) assay, (6 nt: primer-1; 7 nt: primer-2; 8 nt: primer-3; 9 nt: primer-4).

In the novel fluorescent aptamer sensor designed in this paper, whether the primer chain can effectively hybridize and bind with the target triggered open H sequence was the key to the success of this sensor. On the one hand, if the primer sequence is too short, even in the presence of CEA, the hybridization of the primer chain with the opened H-probe is unstable, and it is difficult to trigger the exponential amplification reaction and finally obtain a very low signal-to-noise ratio. On the other hand, if the primer sequence is too long, the primer may open the H-probe even in the absence of the target, thus triggering an exponential amplification reaction, resulting in a very high background signal. To obtain the best signal-to-noise ratio, we designed four primer chains with different numbers of complementary bases to the H-probe and examined the effect of primer chain length on CEA (1 pg/mL) detection. Figure 4 shows the effect of primer chains (primer-1, primer-2, primer-3 and primer-4) with the number of complementary bases to the H-probe between 6 and 9 on the fluorescence intensity. As can be seen from Figure 4, the highest signal-to-noise ratio was obtained for the detection of CEA when the number of bases of the complementary sequence was 8, i.e., when primer-3 was used for the experiments. Thus, the primer-3 was selected as the optimal primer chain and used for the subsequent CEA detection analysis.

### 3.5. Study on the Specificity of CEA Identification

**Figure 5:**
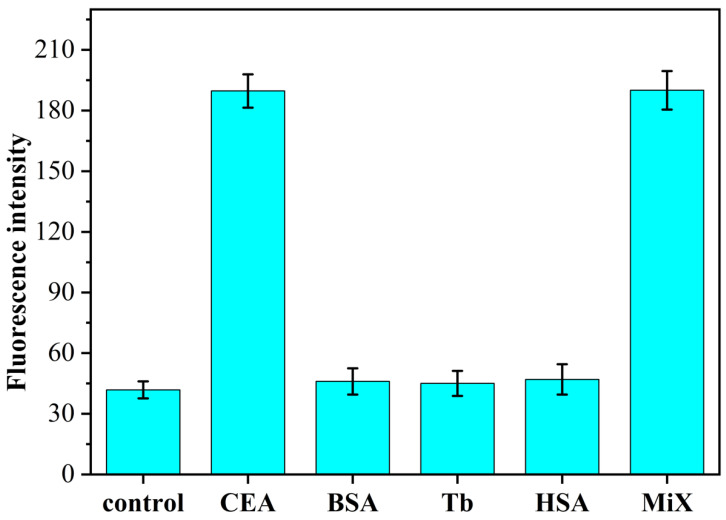
Fluorescence spectrum of H/primer-3/KF/Nb.BbvCI/MB sensor system with CEA specific detection (CEA: 1.0 pg/mL; BSA, Tb and HAS: 1.0 ng/mL).

To investigate the specificity of the constructed fluorescent aptamer sensor for CEA detection, we adopted the H/primer-3/KF/ Nb.BbvCI/MB sensing system for the target CEA (1 pg/mL), the other three non-specific proteins BSA, Tb and HSA (all 1 ng/mL) and their mixtures (CEA was 1 pg/mL, the other proteins was 1 ng/mL) were fluorometrically determined. The results are shown in Figure 5. When other nonspecific proteins (all 1 ng/mL) were added to the H/primer-3/KF/Nb.BbvCI/MB sensing system, the resulting fluorescence intensities did not change significantly compared with those in the absence of CEA presence. When the target protein CEA (1 pg/mL) was added to this sensing system, the fluorescence intensity of this system increased significantly. In order to investigate whether the sensing system has any effect on the detection of CEA in the presence of other proteins, experiments were designed to show that the fluorescence intensity of the sensor also increased significantly when a mixture of CEA and other non-specific proteins was added simultaneously, and the intensity values were almost the same as those obtained when CEA was detected alone. The experimental results show that the fluorescence aptamer sensing method based on isothermal exponential amplification reaction and DNAzyme constructed in this paper has good selectivity and can be used for the specific detection of CEA in serum. When the other non-specific proteins (all 1 ng/mL) were added to the H/primer-3/KF/Nb.BbvCI/MB sensing system, the resulting fluorescence intensity did not change significantly compared with that in the absence of CEA presence. Upon addition of the target protein CEA (1 pg/mL) to this sensing system, the fluorescence intensity of this system increased significantly. Then, in order to investigate whether the sensing system has any effect on the detection of CEA in the presence of other proteins, the experiments were designed to significantly increase the fluorescence intensity of the sensor when CEA and other non-specific protein mixtures were added at the same time, and the intensity value was almost consistent with the fluorescence intensity obtained when CEA was detected alone. The proposed fluorescence aptamer sensing method based on isothermal exponential amplification reaction and DNAzyme has good selectivity and can be used for the specific detection of CEA in serum.

### 3.6. Range of Linearity and Limit of Detection (LOD)

**Figure 6:**
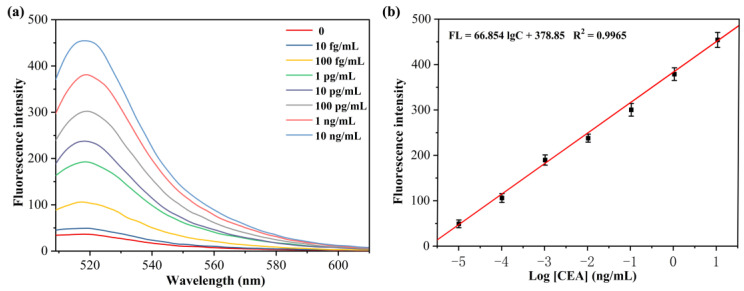
(**a**) Fluorescence spectra of the H probe/primer−3/MB/KF/Nb.BbvCI solution after incubation with different concentrations of CEA. (**b**) Linear calibration curve of FL intensity at 520 nm versus the concentration of CEA.

For investigating the sensitivity of the fluorescence aptamer sensor study based on isothermal exponential amplification reaction and DNAzyme for CEA detection, the relationship between the concentration of CEA and fluorescence intensity was examined by measuring the fluorescence intensity of the sensor after reacting with different concentrations of CEA. The experimental results are shown in Figure 6a. As the concentration of CEA increased, the fluorescence signal of the sensing system was gradually enhanced. This is because the CEA concentration increases, the exponential amplification reaction increases, producing more DNAzyme sequences, which in turn cleaves more MB substrates, and thus the fluorescence signal was stronger. When the linear regression analysis was performed on the logarithmic values of fluorescence intensity values (520 nm) and CEA concentration of the sensing system, a good linear relationship was found for CEA concentration in the range of 10 fg/mL to 10 ng/mL (Figure 6b) with a linear equation: FL = 66.854 lgC + 378.85 (R^2^ = 0.9965) and a detection limit of 5.2 fg/mL for CEA. The results indicate that the proposed sensor can be used for the CEA protein sensitive detection.

### 3.7. Sensor for Human Serum Sample Assay

To investigate whether the fluorescence aptamer sensing method developed in this paper can be used for the analysis of complex samples, six human serum samples were analyzed using this sensing method. These samples were normal human serum samples from 1 to 3 and colon cancer patient serum samples from 4 to 6. All serum samples were diluted 100 times and then subjected to experiments. Fluorescence measurements were performed under optimized experimental conditions and CEA content was calculated. The results of this method were compared with those of the enzyme-linked immunoassay (ELISA) (Table 2). As shown in the results found in Table 2, the CEA determination in human serum samples by the fluorescence aptamer sensing method used in this paper were consistent with the results determined by ELISA. To further investigate the accuracy of the fluorescence aptamer sensing method, we performed spiked recovery tests on these serum samples and found that the recoveries of CEA ranged from 93.5% to 102.4%. The above results indicated that our established fluorescence aptamer sensing method based on isothermal exponential amplification reaction and DNAzyme can be used for the analysis of complex biological samples.

## 4. Conclusions

In summary, the novel ultra-sensitive fluorescent aptamer sensor was constructed and used for the analysis of proteins in blood by utilizing the principles of target-mediated nucleic acid aptamer structure conversion triggering isothermal exponential amplification reaction and DNAzyme signal amplification. The sensing method has the following advantages: Firstly, the sensor exhibits high sensitivity and can detect target protein levels down to fg/mL. Secondly, the aptamer sensor has a wide detection range with a linear span of up to six orders of magnitude. Thirdly, the method has good selectivity and can be used for real biological sample analysis. Accordingly, the sensor is based on signal amplification and can be used for highly selective detection of ultra-low levels of proteins.

## Figures and Tables

**Table 1:** Oligonucleotide sequences (5′→3′).

Oligonucleotides	Sequences
H	ATGCTTGGTACATGGGTGATCGCTGTCGGTATTCCTCAGCCA***ATA*** ***CCAGCTTATTCAATT***CCAGCTCCTCAGCGTATACTGGAATTGAATC
MB	FAM-CACCACTACAAATTATGCTTGGTTrAGGTCGGTATACGAGC GTGTGGTG-DABCYL
DNA-1	TGAGGAATACCGACAGCGATCACCCATGTACCAAGCAT
Primer-1	GATTCA
Primer-2	GATTCAA
Primer-3	GATTCAAT
Primer-4	GATTCAATT

**Table 2:** Results of CEA in human serum samples.

Sample	Present Method (ng/mL)	ELISA (ng/mL)	Relative Deviation (%)
1	5.64	5.40	−4.4
2	3.58	3.32	−7.8
3	4.72	4.58	−3.1
4	206.20	203.00	−1.6
5	186.55	188.34	1.0
6	292.85	289.75	−1.1

## Data Availability

Not applicable.
